# Letter from the Editor in Chief

**DOI:** 10.19102/icrm.2019.100502

**Published:** 2019-05-15

**Authors:** Moussa Mansour


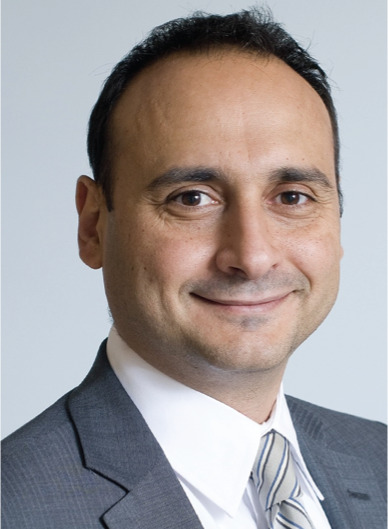


Dear Readers,

This issue of *The Journal of Innovations in Cardiac Rhythm Management* contains a number of important articles covering various areas of electrophysiology practice that are expected to receive wide coverage at the annual scientific meeting of the Heart Rhythm Society happening in San Francisco, CA later this month. Among these topics is atrioesophageal fistula (AEF), one of the most feared complications of atrial fibrillation (AF) ablation. The article on the subject, by Bodziock et al. and titled “Prevention and Treatment of Atrioesophageal Fistula Related to Catheter Ablation for Atrial Fibrillation,”^1^ is a well-written review that covers the mechanisms, prevention, and treatment of this complication.

Despite all of the precautions taken by operators during AF ablation, including reducing the power, contact force, and duration of energy delivery, AEF still occurs. In fact, it is even possible that we might witness an increase in its incidence in the future because of the development of more powerful ablation tools that are aimed at reducing the chance of gap formation along ablation areas and which promote irreversible cardiac tissue injury. At this time, in addition to reducing the ablation parameters while ablating at the posterior left atrial wall, the most commonly used technique for esophageal protection is temperature measurement. Different tools have been developed for this need, including single-sensor probes, multiple-sensor probes, and infrared heat imaging of the esophageal wall. However, temperature measurement for esophageal protection, while helpful, does not constitute a complete solution for the problem. In order to accomplish the creation of irreversible cardiac gap-free ablations, the operator may in some cases need to ablate areas overlying the esophagus, raising the risk of AEF.

In light of the above, mechanical deviation of the esophagus is being increasingly used as a means to protect such during ablation. A few tools have been developed to allow for the deviation to occur and the preliminary results achieved to date are very encouraging. Movement of the esophagus by more than 1 cm to 2 cm away from the ablation area has been demonstrated with some of these tools. More importantly, mechanical deviation of the esophagus does not appear to result in significant complications. As data demonstrating the benefit of this technology remain limited, I expect that randomized studies will be conducted in the near future.

I hope that you enjoy reading this issue and that you find its content useful in your clinical practice.

Best wishes for an enjoyable and productive Heart Rhythm Society meeting.

Sincerely,


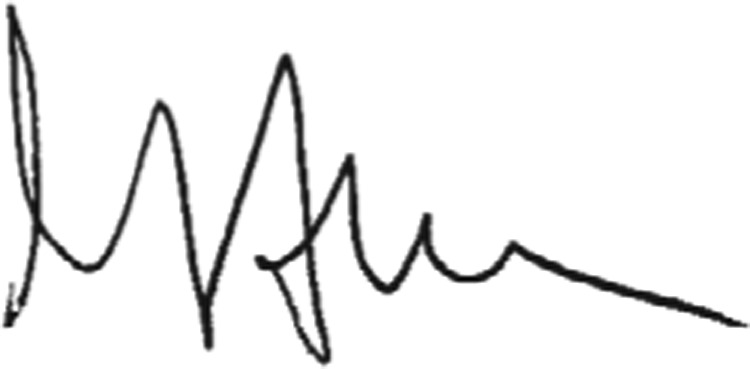


Moussa Mansour, MD, FHRS, FACC

Editor in Chief

The Journal of Innovations in Cardiac Rhythm Management

MMansour@InnovationsInCRM.com

Director, Atrial Fibrillation Program

Jeremy Ruskin and Dan Starks Endowed Chair in Cardiology

Massachusetts General Hospital

Boston, MA 02114
